# Best Practices in Nuclear Imaging for the Diagnosis of Transthyretin Amyloid Cardiomyopathy (ATTR-CM) in KSA: The Eagle Eyes of Local Experts

**DOI:** 10.3390/diagnostics14020212

**Published:** 2024-01-18

**Authors:** Abdullah Alqarni, Ahmed Aljizeeri, Aquib Mohammadidrees Bakhsh, Hossam Ahmed Maher El-Zeftawy, Hussein R. Farghaly, Mukhtar Ahmed M. Alqadhi, Mushref Algarni, Zain Mohammed Asiri, Ahmed Osman, Haya Haddadin, Islam Alayary, Mouaz H. Al-Mallah

**Affiliations:** 1 Prince Sultan Military Medical City, Riyadh 12233, Saudi Arabia; aoalqarni@psmmc.med.sa (A.A.); hfarghaly@psmmc.med.sa (H.R.F.); 2 King Abdulaziz Cardiac Center, Ministry of the National Guard Health Affairs, Riyadh 14626, Saudi Arabia; aljizeeri@yahoo.com; 3 College of Medicine, King Saud bin Abdulaziz University for Health Sciences, Riyadh 11481, Saudi Arabia; 4 King Abdullah International Medical Research Center, Riyadh 21423, Saudi Arabia; 5 Nuclear Medicine Department, King Abdullah Medical City, Makkah 24246, Saudi Arabia; bakhsh.a2@kamc.med.sa; 6 King Faisal Specialist Hospital and Research Centre, Jeddah 11564, Saudi Arabia; helzeftawy@kfshrc.edu.sa; 7 King Fahad Hospital, Hufof, Al-Ahsa Healthcare Cluster, Al-Ahsa 36441, Saudi Arabia; maalqadhi@moh.gov.sa; 8 King Fahad Military Medical Complex, Dhahran 34313, Saudi Arabia; mushref@kfmmc.med.sa; 9 King Saud Medical City, Riyadh 11411, Saudi Arabia; z.assiri@ksmc.med.sa; 10 Pfizer Inc., Riyadh 13244, Saudi Arabia; ahmed.osman3@pfizer.com (A.O.);; 11 Pfizer Gulf FZ LLC, Dubai 29553, United Arab Emirates; haya.haddadin@pfizer.com; 12 Houston Methodist, Weill Cornell Medical College, Houston, TX 77030, USA

**Keywords:** ATTR-CM, cardiac amyloidosis, diagnosis, nuclear imaging, transthyretin

## Abstract

Transthyretin amyloid cardiomyopathy (ATTR-CM) is a complex and serious form of heart failure caused by the accumulation of transthyretin amyloid protein in the heart muscle. Variable symptoms of ATTR-CM can lead to a delayed diagnosis. Recognizing the diagnostic indicators is crucial to promptly detect this condition. A targeted literature review was conducted to examine the latest international consensus recommendations on a comprehensive diagnosis of ATTR-CM. Additionally, a panel consisting of nuclear medicine expert consultants (*n* = 10) and nuclear imaging technicians (*n* = 2) convened virtually from the Kingdom of Saudi Arabia (KSA) to formulate best practices for ATTR-CM diagnosis. The panel reached a consensus on a standard diagnostic pathway for ATTR-CM, which commences by evaluating the presence of clinical red flags and initiating a cardiac workup to assess the patient’s echocardiogram. Cardiac magnetic resonance imaging may be needed, in uncertain cases. When there is a high suspicion of ATTR-CM, patients undergo nuclear scintigraphy and hematologic tests to rule out primary or light-chain amyloidosis. The expert panel emphasized that implementing best practices will support healthcare professionals in KSA to improve their ability to detect and diagnose ATTR-CM more accurately and promptly. Diagnosing ATTR-CM accurately and early can reduce morbidity and mortality rates through appropriate treatment.

## 1. Introduction

Amyloidosis is a rare disease that occurs when amyloid proteins accumulate in organs and tissues of the body. The human heart is frequently impacted by two distinct types of amyloid proteins: the primary or light-chain (AL) and the transthyretin (TTR) amyloidosis proteins [1]. Amyloid fibrils accumulating in the myocardium can lead to heart failure due to infiltrative cardiomyopathy [2,3]. Most patients with TTR amyloidosis have cardiac involvement. TTR is synthesized by the liver and is present in both serum and cerebrospinal fluid. One of its primary functions is to act as a carrier protein for two essential compounds: retinol (vitamin A) and thyroxine (T4) [4,5]. Under normal physiological conditions, TTR is present in the bloodstream in the form of a tetramer that comprises four monomers with beta-sheet rich structures. Changes in the protein’s structure can cause it to lose its tetrameric shape and misfold. As a result, the misfolded TTR can accumulate in different organs of the body and lead to amyloid diseases, such as transthyretin amyloid cardiomyopathy (ATTR-CM) [6]. Invariably, the accumulation of the amyloid protein in various organs of the body can cause other diseases that may hint at the presence of cardiac amyloidosis. Conditions such as carpal tunnel syndrome, lumbar spinal stenosis, bicep tendon rupture, and polyneuropathy affecting the autonomic or sensory systems are associated with amyloidosis [7].

ATTR-CM is classified into two types: hereditary (hATTR-CM) and wild-type (wtATTR-CM). hATTR-CM is a genetic disorder caused by a mutation in the transthyretin gene. On the other hand, wtATTR-CM is a condition that occurs due to aging. In both forms, the transthyretin protein becomes structurally unstable, leading to its accumulation in the heart [8]. The onset of hATTR-CM can occur in people in their 50s to 60s, whereas wtATTR-CM typically develops in older individuals, usually in their late 70s to 80s [8]. The detection of ATTR-CM has frequently been overlooked or delayed because of inadequate diagnostic methods [9].

The diagnostic methods and clinical management strategies of cardiac amyloidosis have undergone significant and impactful changes in the last decade. Previously considered rare due to delayed diagnosis, recent non-biopsy diagnostic advancements have raised awareness of ATTR-CM. In screened patient cohorts, ATTR-CM has been detected in 16% of severe aortic stenosis cases and 13% of heart failure with preserved ejection fraction (HFpEF) cases [10,11]. In France, the incidence of new cases of ATTR-CM increased from 0.6/100,000 person-years in 2011 to 3.6/100,000 person-years in 2019 [12]. Currently, ATTR-CM affects approximately 3 in 10,000 people in Europe [13]. In the United States, an estimated 5000–7000 new cases are diagnosed annually [8].

Despite the progress made in non-invasive diagnostic techniques, identifying cardiac amyloidosis remains a challenging task. Typically, it entails multiple hospital visits and consultations with over five different medical specialties, resulting in an average delay of 8 months in reaching a cardiac amyloidosis diagnosis [14]. This extended timeframe is in part due to the condition’s multi-system effects and the fact that many treating physicians have a low level of suspicion regarding it.

There is a noticeable lack of comprehensive data on the prevalence and incidence of ATTR-CM, in Saudi Arabia and in the neighboring Gulf countries. A survey conducted among 320 physicians in the Gulf region (Bahrain, Kuwait, Oman, and the United Arab Emirates) showed a low level of suspecting the diagnosis among participants despite a high level of perceived awareness about cardiac amyloidosis [15]. The survey also revealed knowledge gaps among physicians regarding non-invasive modalities for diagnosing cardiac amyloidosis [15]. Although most participants found echocardiogram (ECHO) and cardiac magnetic resonance (CMR) imaging useful (82.0% and 91.9%, respectively), only 60% of the participants considered cardiac scintigraphy with bone-avid radio-tracers useful, and one third were unsure about its role [15]. In addition, it is noteworthy that less than half of the participants considered conducting laboratory tests to rule out AL amyloidosis as an important step in the process of the diagnosis. A survey of 85 cardiologists from Saudi Arabia, Egypt, Lebanon, and Iraq revealed that only 71% of the respondents had participated in educational meetings on amyloid cardiomyopathy in the last three years. These findings underscore the importance of raising awareness and standardizing the diagnostic process for cases with suspected cardiac amyloidosis [16]. This article presents the best practices for diagnosing ATTR-CM in the Kingdom of Saudi Arabia (KSA), formulated during an advisory board meeting following a secondary literature review.

## 2. Materials and Methods

Invitations were sent to the leading nuclear laboratories with high volume in all the regions of Saudi Arabia to participate in the literature review and in establishing a best practices statement. Subsequently, a panel consisting of nuclear medicine expert consultants (*n* = 10) and nuclear imaging technicians (*n* = 2) was formed. A targeted literature review was conducted to review the current international consensus recommendations on a comprehensive diagnosis of ATTR-CM. We searched the PubMed, Embase, and Cochrane Library databases for articles published from January 2010 to March 2023 using the following keywords: “transthyretin amyloid cardiomyopathy”, “ATTR-CM”, “diagnosis”, “consensus”, “recommendations”, and “guidelines”. Expert consensus recommendations from the European Society of Cardiology (ESC) and the American Society of Nuclear Cardiology (ASNC) were consulted for the diagnostic pathway for ATTR-CM [2,17]. Additionally, other publications on various aspects of diagnosing ATTR-CM, such as a suspicion of the condition, the use of bone scintigraphy, imaging procedures, imaging parameters, and interpretation of the results, were also referred [3,18,19,20,21,22,23,24,25].

An advisory board meeting was held on 12 April 2023, virtually, by the panel to discuss the findings of the secondary literature review and to agree on the best practices to be adapted for diagnosing ATTR-CM in the KSA. The discussion primarily revolved around several key areas. These included creating a standardized patient referral system for ATTR-CM diagnosis in KSA, assessing the accessibility of nuclear imaging resources across the country, delving into the technical intricacies of nuclear imaging, and tackling the specific challenges related to its utilization in diagnosing ATTR-CM. These discussions were enriched by insights from local experiences in various regions of Saudi Arabia.

## 3. Results and Discussion

ATTR-CM diagnosis is often delayed due to multi-system involvement. Therefore, a timely diagnosis requires the attention of the treating physicians to subtle signs of amyloidosis and the prompt referral of patients with a high likelihood of having ATTR-CM [7].

**Figure 1:**
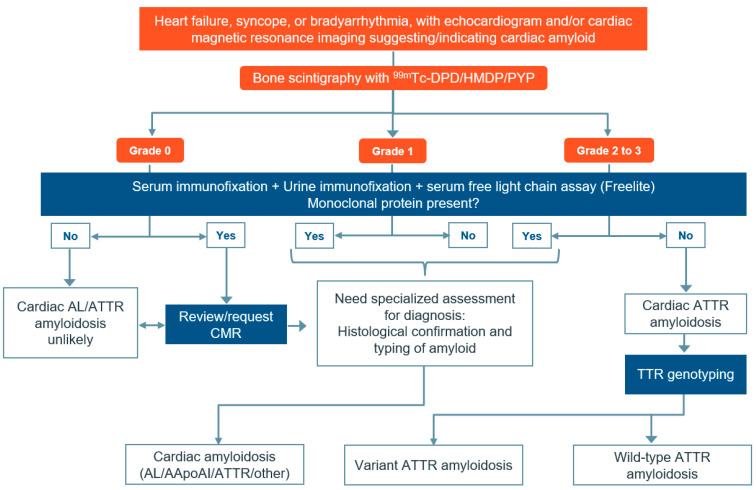
Algorithm for diagnosing amyloid cardiomyopathy in patients with suspected symptoms. Cardiac amyloidosis can be diagnosed by identifying certain echocardiographic features such as increased left ventricular wall thickness, restrictive filling pattern, abnormal left and right ventricular longitudinal strain, and atrial septal thickening. CMR imaging can also be used to detect features of cardiac amyloidosis such as restrictive morphology, abnormal gadolinium kinetics, and extra-cellular volume expansion based on T1 mapping. Abbreviations: AApoA-I: apolipoprotein A-I; AL: light chain amyloidosis; ATTR-CM: transthyretin amyloidosis; CMR: cardiac magnetic resonance imaging; DPD: 3,3-diphosphono-1,2-propanodicarboxylic acid; HDMP: hydroxymethylene diphosphonate; PYP: pyrophosphate; TTR: transthyretin. Source: Rauf et al. 2023 [26].

The diagnostic pathway of ATTR-CM requires a systematic approach, which begins with setting up a high level of clinical suspicion based on the patient’s detailed history and their specific clinical presentation. This is followed by initiating a cardiac workup to assess the patient’s ECHO and CMR imaging results (Figure 1).

### 3.1. ATTR-CM Presentation

Given the range of cardiac and extra-cardiac symptoms associated with ATTR-CM, it is crucial for clinicians to be cognizant of common disease patterns, additional clues, and populations that are commonly affected to ensure an effective diagnosis [27,28]. The most prevalent symptoms of ATTR-CM are those of heart failure, which can be either with preserved or reduced ejection fraction. Additionally, atrial fibrillation is also a common symptom [27,28]. Unexplained peripheral neuropathy or gastrointestinal symptoms are the most common extra-cardiac systemic manifestations of amyloidosis. Additionally, a history of bilateral carpal tunnel syndrome or bicep tendon rupture can increase suspicion of the disease [28].

### 3.2. Clinical Suspicion

Given that the clinical manifestations of ATTR-CM can be non-specific, maintaining a high index of suspicion is crucial for making an accurate diagnosis. The expert panel suggested that it is crucial to comprehend the challenges that patients encounter in their journey to facilitate an early diagnosis, and they drew attention to the inadequate awareness regarding ATTR-CM in primary and secondary healthcare centers. The panel emphasized that coordination and communication between the cardiac centers and the referring personnel are important for an effective diagnosis.

The diagnostic process for ATTR-CM begins with a clinical history and examination, followed by ECHO and CMR imaging [7]. ECHO can help identify diagnostic clues such as increased left ventricular wall thickness (≥12 mm), atrioventricular valve/right ventricle free wall/interatrial septum thickening, diastolic dysfunction, decreased mitral annular systolic velocity, and biatrial enlargement [11,29]. Strain imaging is important, and the apical sparing pattern could be a clue that helps general cardiologist establish the diagnosis. Additionally, the tissue Doppler of the mitral annulus is important. CMR imaging may be necessary for certain patients when ECHO images are suboptimal or when other potential diagnoses, often referred to as “mimickers,” are being considered. CMR can help diagnose ATTR-CM by detecting certain features such as an expansion of the extra-cellular volume, abnormal gadolinium contrast kinetics, and diffuse late gadolinium enhancement [17,30]. Although CMR is an effective tool to rule out amyloidosis in suspected cases of cardiac amyloidosis, it is important to note that it cannot be used as a standalone test. Furthermore, it is neither necessary in all cases nor sufficient for confirming the presence of ATTR-CM or AL amyloidosis as it is unable to distinguish between the two types of cardiac amyloidosis [24,31]. The panel emphasized the importance of a multi-modality diagnostic imaging approach, with an emphasis on ECHO, strain imaging, and CMR imaging in the diagnosis process.

The current literature provides an established diagnostic framework for ATTR-CM, as well as detailing the identification of several red flags that can raise suspicion of ATTR-CM (Table 1). The initial step to suspect ATTR-CM involves using ECHO to evaluate myocardial wall thickness. Further evaluation is recommended for patients who present with heart failure symptoms or red flag symptoms and have a left ventricular wall thickness of ≥12 mm [9,25,27]. The expert panel also opined that the presence of at least one of the red flags and a left ventricular wall thickness ≥12 mm are the general criteria for suspecting ATTR-CM and proceeding for further confirmation through specific testing. ATTR-CM clinical clues/red flags can be categorized into cardiac and extra-cardiac manifestations. Cardiac manifestations of ATTR-CM include increased left ventricular wall thickness in the absence of hypertension or valvular heart disease, heart failure symptoms, diastolic dysfunction, atrial fibrillation, conduction system disease, and elevated cardiac biomarkers. Extra-cardiac manifestations include a history of bilateral carpal tunnel syndrome, spinal stenosis, hip or knee replacement, prior shoulder surgery, proteinuria, or peripheral/autonomic neuropathy [2,3,22,32]. The rationale for suggesting the diagnostic tests and criteria for ATTR-CM is based on the expert consensus, as well as the sensitivity and specificity of the tests.

### 3.3. Diagnosis

The diagnostic confirmation of ATTR-CM requires multiple imaging modalities and may require invasive procedures such as endomyocardial biopsy [11,33]. However, recent developments in non-invasive imaging have made endomyocardial biopsy less essential [34]. A combination of serum/urine electrophoresis and immunofixation tests, along with nuclear scintigraphy findings, is used to diagnose ATTR-CM and rule out AL amyloidosis [20,23,27]. While nuclear scintigraphy has become a crucial diagnostic tool for ATTR-CM, it is important to note that this method alone cannot distinguish between ATTR-CM and AL amyloidosis [28]. ATTR-CM and AL amyloidosis can be differentiated by concomitant testing for light chains [7,23]. Serum/urine electrophoresis and immunofixation may be requested concurrently for nuclear amyloid scan order or before it. An elevated serum light chain should prompt a consultation with a hematology specialist to rule out AL amyloidosis or monoclonal gammopathy of undetermined significance.

#### Nuclear Scintigraphy for Diagnosing ATTR-CM

In contemporary medicine, nuclear scintigraphy is the mainstay for non-invasively diagnosing ATTR-CM. During nuclear scintigraphy, a bone-avid radio tracer known for its affinity to bind amyloid fibrils is administered intravenously. Subsequently, images are acquired using a combination of SPECT (single photon emission computed tomography) and low-dose CT for radio-tracer uptake localization. These images reveal the distribution of the radio tracer within the myocardium. The physician evaluates the images for abnormal radio-tracer uptake in the myocardium, particularly the left ventricular wall. Increased radio-tracer uptake in these areas is suggestive of ATTR amyloid deposition. Semi-quantitative and quantitative analysis techniques may also be employed to assess the extent and severity of myocardial radio-tracer uptake. This can involve calculating ratios such as heart-to-contralateral lung (H/CL) ratios or myocardium-to-background ratios to provide a more objective measurement of amyloid deposition.

Bone-avid radio-tracers such as Tc-99m-DPD (3,3-diphosphono-1,2-propanodicarboxylicacid), Tc-99m-HMDP (hydroxy methylene diphosphonate), and Tc-99m-PYP (pyrophosphate) are commonly used for nuclear scintigraphy, due to their high sensitivity and specificity for ATTR-CM [21]. Tc-99m-PYP is the most preferred radio tracer used in Saudi Arabia, while HMDP has been used in times of Tc-99m-PYP shortages. Nuclear scintigraphy is usually ordered after ECHO. However, in a few rare situations, PYP can be performed without ECHO if other red flags or criteria are met (for example bilateral carpal tunnel syndrome with positive histology for transthyretin amyloidosis, or a positive genotype for common ATTR mutations). The panel also advised that the most preferred radio tracer for screening is PYP (nuclear amyloid scans) and emphasized that it can be used judiciously for cases with clinical suspicion following the use of ECHO and/or CMR. The panel stressed the importance of screening patients with ECHO before proceeding with nuclear imaging.

**Figure 2:**
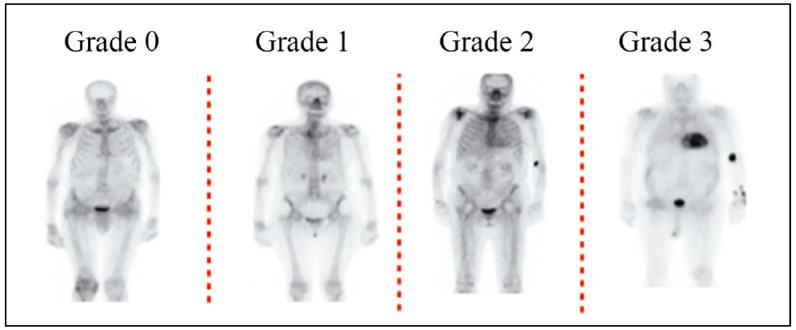
Grading of cardiac absorption in scintigraphy using bisphosphonate. Grade 0: no myocardial uptake of the tracer and standard bone absorption; Grade 1: myocardial absorption of the tracer is less than that of the bone; Grade 2: equal uptake of the tracer by the myocardium and bone; Grade 3: myocardial uptake surpasses bone uptake, with diminished or no bone absorption. Sources: Perugini et al. 2005 [35]; Garcia-Pavia et al. 2021 [38]; Chander Mohan et al. 2022 [22].

The nuclear scans should be performed in accordance with the ASNC protocol for cardiac nuclear imaging, with slight modifications as per the patient’s needs and the available infrastructure [17]. Almost all the scans are undertaken with SPECT imaging for the localization of the tracer uptake within the myocardium [17]. The images acquired through bone scintigraphy for the diagnosis of ATTR-CM can be analyzed by semi-quantitative and quantitative methods. The Perugini grading system is used to semi-quantitatively assess cardiac uptake, which is divided into four categories: Grade 0 indicates no visible cardiac uptake, Grade 1 indicates mild cardiac uptake visible but inferior to skeletal (rib) uptake, Grade 2 indicates moderate cardiac uptake visible equal to or greater than skeletal (rib) uptake, and Grade 3 indicates strong cardiac uptake with little or no skeletal (rib) uptake [34,35,36]. An uptake of Grade 2 and above is considered significant; scans with Grades 2 and 3 have a positive predictive value of 100% for detecting ATTR-CM with a specificity of 87% and sensitivity of 97% (Figure 2). For Grade 1, histological confirmation (cardiac or extra-cardiac) is required [22,37].

Quantitative assessment involves comparing counts from a region of interest situated over the heart to a region of comparable intensity located on the chest’s opposite side. For all scans that test positive, SPECT (or SPECT/Computed Tomography [CT]) is evaluated to ensure that the uptake signifies the tracer’s retention in the myocardium and not a signal from the blood pool. This is especially crucial in patients with low cardiac output, as the radio tracer may remain in the blood pool [19,39,40].

For an accurate diagnosis, it is crucial to exclude AL amyloidosis while identifying ATTR-CM, given that there is a risk of up to 13% for false positive outcomes in patients suffering from AL amyloidosis [22,23]. To achieve this, hematologic assessments, such as serum free light chain (FLC) assay (Kappa/Lambda Ratio), serum protein electrophoresis with immunofixation (SPIE), and urine protein electrophoresis with immunofixation (UPIE), are often used in combination with nuclear scintigraphy, for a comprehensive diagnostic evaluation of ATTR-CM. The FLC assay is helpful in ruling out AL amyloidosis, as an abnormal Kappa/Lambda ratio suggests the presence of clonal plasma cell disorders, which are indicative of AL amyloidosis or monoclonal gammopathy of undetermined significance. A normal Kappa/Lambda ratio supports the possibility of ATTR-CM as the underlying cause. SPIE can help detect and characterize abnormal protein bands that may indicate the presence of monoclonal proteins associated with AL amyloidosis or other systemic conditions. UPIE is like SPIE but focuses on protein analysis in urine. It aids in identifying abnormal protein bands in the urine, which can be indicative of AL amyloidosis or other underlying conditions. Employing both serum and urine immunofixation in conjunction with the FLC assay offers a high level of precision (with a sensitivity of 99%) in identifying the occurrence of AL amyloidosis [22,37]. The expert panel suggested that the timing of hematologic assessments, whether before or along with bone scintigraphy, should be up to the referring physician’s discretion. Histologic confirmation using techniques like immunohistochemistry or mass spectrometry is essential to find out the type of amyloid deposits when AL amyloidosis is suggested but not confirmed or ruled out by bone scintigraphy and monoclonal protein tests [2,22,32].

The expert panel opined that it is equally important to recognize the causes of false positive results, such as acute myocardial infarction, that can have higher uptake that resembles amyloidosis. The need for endomyocardial biopsy should be left to the discretion of the treating physician. It should be considered in cases with very high clinical, ECHO, and MRI findings suggesting cardiac amyloidosis with a negative PYP scan and in cases of equivocal PYP scan results with still high clinical suspicion. It is also important to properly select patients referred for Tc-99m-PYP scans, particularly in busy laboratories and during Tc-99m-PYP shortages.

## 4. Best Practices Recommended by the Expert Panel

The panel agreed on a set of recommendations in the following situations:

### 4.1. Suspicion of ATTR-CM

- Considering the multi-organ involvement of ATTR-CM, an interdisciplinary strategy is required involving collaboration between nuclear medicine specialists, cardiologists, and other healthcare professionals is recommended for assessment and diagnosis.
- The patient evaluation should start with an assessment of medical history, including symptoms and risk factors for ATTR-CM. Further, the patient evaluation should include a thorough physical examination to assess for signs of cardiac involvement, such as heart murmurs, abnormal heart sounds, and signs of fluid retention.
- Patients should be screened for any clinical features suggestive of the phenotype of ATTR-CM, particularly those with a constellation of cardiac, neurological, and musculoskeletal manifestations.
- A transthoracic echocardiogram (TTE) should be conducted evaluating cardiac structure and function. One should look for signs of cardiac hypertrophy, thickened myocardium, and abnormal diastolic function, which are common findings in ATTR-CM. TTE can often help differentiate between restrictive and hypertrophic cardiomyopathies.
- Patients exhibiting an enlarged ventricular wall thickness (≥12 mm), along with at least one of the following indicators or warning signs, should be evaluated for ATTR-CM: an apical sparing longitudinal strain pattern, orthopedic symptoms (such as bilateral carpal tunnel syndrome, lumbar spinal stenosis, or a rupture of the bicep tendon), consistently elevated troponin levels, atrioventricular conduction block, or peripheral or autonomic neuropathy.
- In instances where the TTE is of subpar quality or yields indeterminate results, CMR becomes necessary. Diagnostic characteristics of CMR encompass an enlargement of the extra-cellular volume, irregular kinetics of gadolinium contrast (imperfect nulling), and widespread late gadolinium enhancement.
- Red flags, ECG, and ECHO can be employed to raise suspicions of amyloidosis. Nuclear scintigraphy may be considered to differentiate ATTR-CM and AL.
- Hematologic tests should be carried out to rule out AL amyloidosis either before or at the time of ordering PYP imaging. An abnormal hematologic test should trigger a hematology consultation.

### 4.2. Diagnosing ATTR-CM

The confirmation of ATTR-CM is based on the finding of the Tc-99m-PYP scan and hematologic tests to rule out cardiac involvement with AL amyloidosis. The following are the recommendations of the panel regarding Tc-99m-PYP image acquisition. The technical aspects of bone scintigraphy for the detection of ATTR-CM are adapted from ASNC practice points for the imaging of amyloidosis [17].

- Imaging procedures:
  ○ No special preparation or fasting is required for the patient undergoing bone scintigraphy.
  ○ Rest scan is preferred using the radio tracer, 99mTc-PYP at a10–20 mCi/370–740 MBq (intravenous) dose; HMDP can be used in case of shortages of PYP.
  ○ Time between injection and acquisition:
    ▪ Depending on the patient’s uptake and various other factors, 2 h or 3 h is recommended. One hour interval (only with 99mTc-PYP) is optional. If any excess blood pool activity is observed on the 1 h images, then imaging after a 3 h interval is recommended.
    ▪ For elderly or intolerant patients, if results are conclusive in 1 h, consider stopping imaging instead of proceeding for 3 h.
  ○ When available, SPECT/CT imaging is preferred for radio-tracer localization.
- Imaging parameters:
  ○ Suggested cardiac or chest field of view with SPECT and planar imaging in supine position.
  ○ Suggested other imaging parameters include:
    ▪ Energy window: 140 keV, 15–20%.
    ▪ Collimators: Low energy, high resolution.
    ▪ Matrix: Planar: 256 by 256; at least 64 by 64 is required. SPECT: 128 by 128; at least 64 by 64 is required.
    ▪ Pixel: 2.3–6.5 mm.
- Planar imaging parameters:
  ○ Number of views: anterior, lateral, and left anterior oblique.
  ○ Detector configuration: 90 degrees.
  ○ Image duration (count based): 750,000 counts.
  ○ Magnification: 1.46.
- SPECT imaging parameters:
  ○ Angular range: Recommended: 180 degrees; Optional: 360 degrees.
  ○ Detector configuration: Recommended 90 degrees; Optional 180 degrees.
  ○ ECG gating: Off; non-gated imaging.
  ○ Number of views/detectors: 40.
  ○ Time per stop: 20 s.
  ○ Magnification: 1.0.
- Interpretation:
  ○ Visual interpretation:
    ▪ To confirm diffuse radio-tracer uptake in the myocardium, planar and SPECT or SPECT/CT images can be evaluated.
    ▪ To differentiate myocardial radio-tracer uptake from residual blood pool activity, focal myocardial infarct, and overlapping bone (e.g., from rib hot spots from fractures) on SPECT images, planar and SPECT or SPECT/CT images can be evaluated, as per physician’s discretion and accessibility to the technique. If excess blood pool activity is noted on the 1 h SPECT images, repeating SPECT imaging at 3 h is recommended. If myocardial tracer uptake is visually present on SPECT, proceed to semi-quantitative visual grading. If no myocardial tracer uptake is present on SPECT, the visual grade is 0.
    ▪ If myocardial tracer uptake is visually present on SPECT, semi-quantitative visual grading can be performed. If no myocardial tracer uptake is present on SPECT, the visual grade is 0.
  ○ To distinguish between AL and ATTR cardiac amyloidosis, semi-quantitative grading can be performed using planar and SPECT or SPECT/CT images. These images are then examined for any relative tracer uptake in the myocardium in comparison to the ribs. Based on these observations, the images are then graded using the following scale:
    ▪ Grade 0: This grade is assigned when there is no observable myocardial uptake, and the bone uptake appears to be within normal limits.
    ▪ Grade 1: This grade is given when the myocardial uptake is observed to be less than the uptake in the ribs.
    ▪ Grade 2: This grade is assigned when the myocardial uptake is found to be equal to the rib uptake.
    ▪ Grade 3: This grade is given when the myocardial uptake is greater than the rib uptake, and the rib uptake is either mild or not present at all.
  ○ When applicable, heart-to-contralateral lung (H/CL) uptake ratio assessment can be performed to identify ATTR cardiac amyloidosis. H/CL ratios of ≥1.5 at 1 h can accurately identify ATTR cardiac amyloidosis if myocardial pyrophosphate (PYP) uptake is visually confirmed on SPECT and systemic AL amyloidosis is excluded. An H/CL ratio of ≥1.3 at 3 h can also identify ATTR cardiac amyloidosis.
  ○ The detection of ATTR-CM cannot rely solely on H/CL ratio with PYP. H/CL ratio is not recommended if there is an absence of myocardial uptake on SPECT. If there is a discordant result or the visual grade is equivocal, the H/CL ratio may be helpful to classify it as positive or negative. Both H/CL and a semi-quantitative visual system are recommended for assessing cardiac amyloidosis via scintigraphy.

## 5. Conclusions and Future Perspectives

ATTR-CM remains challenging and often goes unrecognized due to its non-specific clinical presentation and lack of awareness among physicians. However, as treatment strategies advance, awareness of the disease is expected to increase. The use of 99mTc-labeled bone radio-tracer scintigraphy has introduced a non-invasive and highly accurate diagnostic method for the early and definitive detection of ATTR-CM. Presently, studies are focused on the development of novel radio-tracers that are specifically designed to target amyloid deposits in the myocardium. This advancement aims to improve the precision of visualization and quantification of amyloid burden [9]. Additionally, the integration of hybrid imaging technologies, such as SPECT/CT or PET/CT, has demonstrated potential in enhancing anatomical localization and aiding in the differentiation of cardiac amyloidosis from other conditions [41]. Moreover, the implementation of quantitative analysis methods and artificial intelligence algorithms has shown promise in streamlining the interpretation of bone scintigraphy images and enhancing diagnostic accuracy [38,41]. These advancements indicate that bone scintigraphy will increasingly play a crucial role in the early and accurate determination of ATTR-CM, enabling timely intervention and improved patient outcomes. We anticipate that the publication of recommendations on the best practices for performing nuclear scintigraphy in the detection of ATTR-CM is crucial. It will likely standardize the protocols among nuclear laboratories and help new laboratories in the setup of amyloidosis scintigraphy services. Improving the imaging protocols will result in a better diagnosis of ATTR-CM.

## Figures and Tables

**Table 1:** Possible signs (red flags) of transthyretin amyloid cardiomyopathy.

Cardiac Signs	Extra-Cardiac Signs
Blood pressure that is low or normal after having hypertension; Irregular and fast atrial rhythm with conduction system disorder; Increased LV wall thickness; Abnormalities of cardiac rhythm and conduction in HFpEF; Impairment of the atrioventricular and sinoatrial nodes; Abnormalities of cardiac rhythm and conduction; Aortic stenosis with low flow and gradient; Diffuse ischemia causing cardiogenic shock (uncommon); Reduced QRS voltage on ECG indicating a false infarct pattern; High NT-proBNP levels relative to HF severity; Persistently elevated troponin levels; Increased valve thickness; Gadolinium enhancement in the innermost layer of the heart wall; Irregular gadolinium kinetics; Increased extra-cellular volume.	Bilateral carpal tunnel syndrome/weakness or paresthesia of hands; Atraumatic biceps tendon rupture, lumbar spinal stenosis; Peripheral neuropathy and dysautonomia; Diarrhea and/or constipation, nausea and vomiting, and early satiety, leading to weight loss; Glaucoma, intravitreal deposition, and scalloped pupils; Hepatomegaly and renal disease.

Abbreviations: ECG, electrocardiogram; HF, heart failure; HFpEF, heart failure with preserved ejection fraction; LGE, late gadolinium enhancement; LV, left ventricular; NTproBNP, N-terminal pro b-type natriuretic peptide; QRS: refers to the graphical deflections seen on the ECG, representing the depolarization of the ventricles. Source: Mohan et al., 2022 [22].

## Data Availability

No datasets were analyzed or generated during the present study.

## References

[1] Benson M.D., Buxbaum J.N., Eisenberg D.S., Merlini G., Saraiva M.J.M., Sekijima Y., Sipe J.D., Westermark P. (2018). Amyloid nomenclature 2018: Recommendations by the International Society of Amyloidosis (ISA) nomenclature committee. Amyloid.

[2] Garcia-Pavia P., Bengel F., Brito D., Damy T., Duca F., Dorbala S., Nativi-Nicolau J., Obici L., Rapezzi C., Sekijima Y. (2021). Expert consensus on the monitoring of transthyretin amyloid cardiomyopathy. Eur. J. Heart Fail..

[3] Lin W., Chattranukulchai P., Lee A.P., Lin Y.H., Yu W.C., Liew H.B., Oomman A. (2022). Clinical recommendations to diagnose and monitor patients with transthyretin amyloid cardiomyopathy in Asia. Clin. Cardiol..

[4] Kelly J.W., Colon W., Lai Z., Lashuel H.A., McCulloch J., McCutchen S.L., Miroy G.J., Peterson S.A. (1997). Transthyretin quaternary and tertiary structural changes facilitate misassembly into amyloid. Adv. Protein Chem..

[5] Liz M.A., Mar F.M., Franquinho F., Sousa M.M. (2010). Aboard transthyretin: From transport to cleavage. IUBMB Life.

[6] Cornwell G.G., Sletten K., Johansson B., Westermark P. (1988). Evidence that the amyloid fibril protein in senile systemic amyloidosis is derived from normal prealbumin. Biochem. Biophys. Res. Commun..

[7] Kittleson M.M., Maurer M.S., Ambardekar A.V., Bullock-Palmer R.P., Chang P.P., Eisen H.J., Nair A.P., Nativi-Nicolau J., Ruberg F.L. (2020). Cardiac Amyloidosis: Evolving Diagnosis and Management: A Scientific Statement From the American Heart Association. Circulation.

[8] Jain A., Zahra F. (2022). Transthyretin Amyloid Cardiomyopathy (ATTR-CM). StatPearls [Internet].

[9] Ruberg F.L., Grogan M., Hanna M., Kelly J.W., Maurer M.S. (2019). Transthyretin Amyloid Cardiomyopathy: JACC State-of-the-Art Review. J. Am. Coll. Cardiol..

[10] González-López E., Gallego-Delgado M., Guzzo-Merello G., de Haro-Del Moral F.J., Cobo-Marcos M., Robles C., Bornstein B., Salas C., Lara-Pezzi E., Alonso-Pulpon L. (2015). Wild-type transthyretin amyloidosis as a cause of heart failure with preserved ejection fraction. Eur. Heart J..

[11] Castaño A., Narotsky D.L., Hamid N., Khalique O.K., Morgenstern R., DeLuca A., Rubin J., Chiuzan C., Nazif T., Vahl T. (2017). Unveiling transthyretin cardiac amyloidosis and its predictors among elderly patients with severe aortic stenosis undergoing transcatheter aortic valve replacement. Eur. Heart J..

[12] Damy T., Bourel G., Slama M., Algalarrondo V., Lairez O., Fournier P., Costa J., Pelcot F., Farrugia A., Zaleski I.D. (2023). Incidence and survival of transthyretin amyloid cardiomyopathy from a French nationwide study of in-and out-patient databases. Arch. Cardiovasc. Dis. Suppl..

[13] Rozenbaum M.H., Large S., Bhambri R., Stewart M., Whelan J., van Doornewaard A., Dasgupta N., Masri A., Nativi-Nicolau J. (2021). Impact of Delayed Diagnosis and Misdiagnosis for Patients with Transthyretin Amyloid Cardiomyopathy (ATTR-CM): A Targeted Literature Review. Cardiol. Ther..

[14] Dang D., Fournier P., Cariou E., Huart A., Ribes D., Cintas P., Roussel M., Colombat M., Lavie-Badie Y., Carrié D. (2020). Gateway and journey of patients with cardiac amyloidosis. ESC Heart Fail..

[15] Al Badarin F., Al-Humood K., Bader F., Alsaid S., Sulaiman K., Alzadjali M., Sabbour H., Shehab A., Bazargani N., Perlini S. (2022). Physician Knowledge and Awareness About Cardiac Amyloidosis in the Middle East and Gulf Region. JACC CardioOncol..

[16] Mohty D., Nasr S., Ragy H., Farhan H.A., Fadel B., Alayary I., Ghoubar M. (2023). Cardiac amyloidosis: A survey of current awareness, diagnostic modalities, treatment practices, and clinical challenges among cardiologists in selected Middle Eastern countries. Clin. Cardiol..

[17] Dorbala S., Ando Y., Bokhari S., Dispenzieri A., Falk R.H., Ferrari V.A., Fontana M., Gheysens O., Gillmore J.D., Glaudemans A. (2021). ASNC/AHA/ASE/EANM/HFSA/ISA/SCMR/SNMMI Expert Consensus Recommendations for Multimodality Imaging in Cardiac Amyloidosis: Part 1 of 2-Evidence Base and Standardized Methods of Imaging. Circ. Cardiovasc. Imaging.

[18] Apostolou E.A., Fontrier A.M., Efthimiadis G.K., Kastritis E., Parissis J., Kanavos P. (2023). The patient pathway in ATTR-CM in Greece and how to improve it: A multidisciplinary perspective. Hell. J. Cardiol..

[19] Singh V., Falk R., Di Carli M.F., Kijewski M., Rapezzi C., Dorbala S. (2019). State-of-the-art radionuclide imaging in cardiac transthyretin amyloidosis. J. Nucl. Cardiol..

[20] Maurer M.S., Bokhari S., Damy T., Dorbala S., Drachman B.M., Fontana M., Grogan M., Kristen A.V., Lousada I., Nativi-Nicolau J. (2019). Expert Consensus Recommendations for the Suspicion and Diagnosis of Transthyretin Cardiac Amyloidosis. Circ. Heart Fail..

[21] Bokhari S., Castaño A., Pozniakoff T., Deslisle S., Latif F., Maurer M.S. (2013). (99m)Tc-pyrophosphate scintigraphy for differentiating light-chain cardiac amyloidosis from the transthyretin-related familial and senile cardiac amyloidoses. Circ. Cardiovasc. Imaging.

[22] Chander Mohan J., Dalal J., Chopra V.K., Narasimhan C., Kerkar P., Oomman A., Ray Fcsi S., Sharma A.R., Dougall P., Simon S. (2022). Suspecting and diagnosing transthyretin amyloid cardiomyopathy (ATTR-CM) in India: An Indian expert consensus. Indian Heart J..

[23] Gillmore J.D., Maurer M.S., Falk R.H., Merlini G., Damy T., Dispenzieri A., Wechalekar A.D., Berk J.L., Quarta C.C., Grogan M. (2016). Nonbiopsy Diagnosis of Cardiac Transthyretin Amyloidosis. Circulation.

[24] Dorbala S., Cuddy S., Falk R.H. (2020). How to Image Cardiac Amyloidosis: A Practical Approach. JACC Cardiovasc. Imaging.

[25] Vergaro G., Aimo A., Barison A., Genovesi D., Buda G., Passino C., Emdin M. (2020). Keys to early diagnosis of cardiac amyloidosis: Red flags from clinical, laboratory and imaging findings. Eur. J. Prev. Cardiol..

[26] Rauf M.U., Hawkins P.N., Cappelli F., Perfetto F., Zampieri M., Argiro A., Petrie A., Law S., Porcari A., Razvi Y. (2023). Tc-99m labelled bone scintigraphy in suspected cardiac amyloidosis. Eur. Heart J..

[27] Witteles R.M., Bokhari S., Damy T., Elliott P.M., Falk R.H., Fine N.M., Gospodinova M., Obici L., Rapezzi C., Garcia-Pavia P. (2019). Screening for Transthyretin Amyloid Cardiomyopathy in Everyday Practice. JACC Heart Fail..

[28] Sabbour H., Hasan K.Y., Al Badarin F., Alibazoglu H., Rivard A.L., Romany I., Perlini S. (2021). From Clinical Clues to Final Diagnosis: The Return of Detective Work to Clinical Medicine in Cardiac Amyloidosis. Front. Cardiovasc. Med..

[29] Longhi S., Lorenzini M., Gagliardi C., Milandri A., Marzocchi A., Marrozzini C., Saia F., Ortolani P., Biagini E., Guidalotti P.L. (2016). Coexistence of Degenerative Aortic Stenosis and Wild-Type Transthyretin-Related Cardiac Amyloidosis. JACC Cardiovasc. Imaging.

[30] Dorbala S., Ando Y., Bokhari S., Dispenzieri A., Falk R.H., Ferrari V.A., Fontana M., Gheysens O., Gillmore J.D., Glaudemans A. (2020). ASNC/AHA/ASE/EANM/HFSA/ISA/SCMR/SNMMI expert consensus recommendations for multimodality imaging in cardiac amyloidosis: Part 2 of 2-Diagnostic criteria and appropriate utilization. J. Nucl. Cardiol..

[31] Brownrigg J., Lorenzini M., Lumley M., Elliott P. (2019). Diagnostic performance of imaging investigations in detecting and differentiating cardiac amyloidosis: A systematic review and meta-analysis. ESC Heart Fail..

[32] Kittleson M.M., Ruberg F.L., Ambardekar A.V., Brannagan T.H., Cheng R.K., Clarke J.O., Dember L.M., Frantz J.G., Hershberger R.E., Maurer M.S. (2023). 2023 ACC Expert Consensus Decision Pathway on Comprehensive Multidisciplinary Care for the Patient With Cardiac Amyloidosis: A Report of the American College of Cardiology Solution Set Oversight Committee. J. Am. Coll. Cardiol..

[33] Zeldenrust S.R., Benson M.D. (2010). Familial and senile amyloidosis caused by transthyretin. Protein Misfolding Diseases: Current and Emerging Principles and Therapies.

[34] Hutt D.F., Fontana M., Burniston M., Quigley A.M., Petrie A., Ross J.C., Page J., Martinez-Naharro A., Wechalekar A.D., Lachmann H.J. (2017). Prognostic utility of the Perugini grading of 99mTc-DPD scintigraphy in transthyretin (ATTR) amyloidosis and its relationship with skeletal muscle and soft tissue amyloid. Eur. Heart J. Cardiovasc. Imaging.

[35] Perugini E., Guidalotti P.L., Salvi F., Cooke R.M., Pettinato C., Riva L., Leone O., Farsad M., Ciliberti P., Bacchi-Reggiani L. (2005). Noninvasive etiologic diagnosis of cardiac amyloidosis using 99mTc-3,3-diphosphono-1,2-propanodicarboxylic acid scintigraphy. J. Am. Coll. Cardiol..

[36] Ren C., Ren J., Tian Z., Du Y., Hao Z., Zhang Z., Fang W., Li F., Zhang S., Hsu B. (2021). Assessment of cardiac amyloidosis with (99m)Tc-pyrophosphate (PYP) quantitative SPECT. EJNMMI Phys..

[37] Maurer M.S., Elliott P., Comenzo R., Semigran M., Rapezzi C. (2017). Addressing Common Questions Encountered in the Diagnosis and Management of Cardiac Amyloidosis. Circulation.

[38] Garcia-Pavia P., Rapezzi C., Adler Y., Arad M., Basso C., Brucato A., Burazor I., Caforio A.L.P., Damy T., Eriksson U. (2021). Diagnosis and treatment of cardiac amyloidosis: A position statement of the ESC Working Group on Myocardial and Pericardial Diseases. Eur. Heart J..

[39] Castano A., Haq M., Narotsky D.L., Goldsmith J., Weinberg R.L., Morgenstern R., Pozniakoff T., Ruberg F.L., Miller E.J., Berk J.L. (2016). Multicenter Study of Planar Technetium 99m Pyrophosphate Cardiac Imaging: Predicting Survival for Patients With ATTR Cardiac Amyloidosis. JAMA Cardiol..

[40] Stern L.K., Kittleson M.M. (2021). Updates in Cardiac Amyloidosis Diagnosis and Treatment. Curr. Oncol. Rep..

[41] Duran J.M., Borges-Neto S. (2023). Bone scintigraphy imaging and transthyretin-related (ATTR) cardiac amyloidosis: New tricks from an old tool?. J. Nucl. Cardiol..

